# Genotype Variations of rs13381800 in TCF4 Gene and rs17039988 in NRXN1 Gene among a Sample of Iranian Patients with Schizophrenia

**Published:** 2019-10

**Authors:** Mohadeseh Agahi, Zahra Noormohammadi, Iman Salahshourifar, Niloufar Mahdavi Hezaveh

**Affiliations:** 1 Department of Biology, Science and Research Branch, Islamic Azad University, Tehran, Iran.; 2 Department of Psychiatry, School of Medicine, Shahid Beheshti University of Medical Sciences, Tehran, Iran.

**Keywords:** *Neurexin 1*, *rs13381800*, *rs17039988*, *Schizophrenia*, *Transcription Factor 4*

## Abstract

**Objective:** Schizophrenia is a complicated mental disorder that affects about 1% of the world's population. It is a complex disease and is approximately 80% inherited. One of the candidate genes in schizophrenia is transcription factor 4 (TCF4), which is positioned on chromosome 18 and is a transcription factor that plays a role in the transcription of Neurexin 1(NRXN1) gene, which is one of the candidate genes for developing schizophrenia. This case-control study aimed to investigate the correlation of TCF4 rs13381800 and NRXN1 rs17039988 polymorphisms with the risk of schizophrenia in a sample of Iranian patients with schizophrenia.

**Method** **:** A total of 200 individuals were included in this study: 100 patients with schizophrenia (65 males and 35 females), with the mean age of 40.80 ± 11.298 years, and 100 as a control group (63 males and 37 females), with the mean age 32.92 ± 7.391 years. Allele specific polymerase chain reaction and restriction fragment length polymorphism (PCR-RFLP) were done, respectively, for genotyping of rs13381800 (T/C) and rs17039988 (A/C) polymorphisms.

**Results: **The results showed that the frequency of C / C genotype in rs13381800 in patients’ group was 9%, while it was 13% in the control group. Also, the frequency of C / C genotype in rs17039988 was 9% in patients and 7% in control groups. Statistical analysis of polymorphisms showed no correlation between patients and controls in rs13381800 (OR = 1.51; CI = 95%; P = 0.366) and rs17039988 (OR = 0.76; CI = 95%; P = 0.602).

**Conclusion: **No significant difference was found between rs13381800 and rs17039988 genotypes between patients and control groups in terms of gender, age and education in the patients group. Our study suggests that there was no correlation between desired polymorphisms with schizophrenia in the studied population.

Schizophrenia (MIM 181500) is a severe psychiatric disorder that occurs in early childhood, and about 21 million people worldwide suffer from this disorder (World Health Organization 2017). Symptoms of this disorder include irregularities of thoughts, feelings and behaviors, hallucinations and delusions, and motility, which make this disorder one of the top 10 causes of disability (1). Some pre- and post-birth factors, including oxygen deficiency and infections, have a significant effect on developing schizophrenia, and men are more likely to develop it (2). Poor educational achievement is associated with schizophrenia (3). The problem with the diagnosis of schizophrenia is that the symptoms of this disorder can overlap with other disorders, such as mania and depression. 

Schizophrenia is a group of disorders that overlap with autism spectrum disorder, bipolar disorder, and schizoaffective disorder. Genetic studies have also confirmed this overlap (4, 5). Many genetic studies have been conducted to understand the etiology of schizophrenia (6). One of the results of these genetic studies was the identification of candidate genes for the development of schizophrenia and their related genetic polymorphisms (7). The incidence of schizophrenia is about 80% related to genetic causes (8). 

Many studies have been conducted to identify common and rare variations that have significant correlations with schizophrenia (9). The genetic etiology of schizophrenia in humans provides the evidence of both the common and rare variants that cause the disorder (8). By April 2014, according to 31 genome wide association studies for schizophrenia, more than 60 polymorphisms involved in the development of schizophrenia with the p-value of <5 x 10-8. The molecular studies performed on the genes determined by GWAS have shown that those genes affect neuronal activity, differentiation, and neuronal maturation in various pathways (10). One of these genes is TCF4 gene (GeneID: 6925), a transcription factor and one of the most commonly identified candidates for schizophrenia, which is located on chromosome 18 (11). TCF4 is expressed mainly in the neocortex and hippocampal regions and has several isoforms and encodes a protein that has a helix-loop-helix motif (bHLH) (9), which is a CANNTG sequence in the regulatory domain of DNA or E-box, which is why it has been called E-protein (12).

Since NRXN1 promoter has a single conserved E-box, TCF4 plays a role in the transcription of the NRXN1 gene by affecting the putative promoter region of NRXN1 (13, 14). Genetic studies have shown that neurexin 1 gene can be considered as a candidate gene for the development of the human brain to cause mental disorders, such as schizophrenia, autism, and mental retardation (15, 16). NRXN1 gene is located on chromosome 2p16.3 and has 23 exons that contain 1.2 Mb of the genome, which is why it is one of the largest genes in the human genome (17). NRXN1 acts as adhesion precursor molecule. Neurexin-1α has been reported to interact with postsynaptic neuroligins, as mediators in synaptic pathways dependent on GABA and glutamate (17). This protein also binds to leucine-rich repeats of proteins that pass through the membrane and play a role in the synapse of glutamate (18). The association between the polymorphisms of the TCF4 gene and NRXN1 gene with schizophrenia has been reported in many studies (19, 20, 21, 22, 23, and 24). However, other studies failed to find a link between the single nucleotide polymorphisms of these 2 genes and schizophrenia (25, 26, 27, and 28).

The role of TCF4 variations in other diseases, such as the rare Pete Hopkins syndrome, intellectual disability, Fuchs endothelial corneal dystrophy, obesity, diabetes, and various types of cancers have been reported (29, 30, 31, 32, and 33). Single nucleotide polymorphisms in NRXN1 gene have also been observed about nicotine dependence and Hirschsprung's disease (34, 35).

Both rs13381800 SNP and rs17039988 SNP are located at promoter flanking regions which are in the upstream of gene promoters and they might have a regulatory rule in gene transcription (36). Since TCF4 protein also binds to NRXN1 gene promoter region, if the expression of this transcription factor is low, it also affects the expression of NRXN1 gene (14). To date, no study has investigated the correlation of these two polymorphisms with the incidence of schizophrenia in an Iranian population. Therefore, in the present study, TCF4 and NRXN1 genes were selected and a correlation study of rs13381800 in the promoter region of TCF4 gene with rs17039988 in the promoter region of NRXN1 gene was performed in samples collected from individuals with schizophrenia and healthy controls.

## Materials and Methods

In this study, 100 (63 males and 37 females) unrelated healthy individuals, with the mean age of 32.92 ± 7.391 years, and 100 (65 males and 35 females) unrelated patients with schizophrenia, with the mean age of 40.80 ± 11.298 years, who were referred to the psychiatric ward of Imam Hossein and 506 Artesh hospitals, were selected to give blood samples. The patients were selected using research tools, including a demographic questionnaire and Positive and Negative Syndrome Scale (PANSS), and a specialist psychiatrist approved the clinical interview based on Diagnostic and Statistical Manual of Mental Disorders, Fourth Edition, Text Revision (DSM-IV-TR). Written consent was obtained from both patients and controls. Healthy controls were selected using PANSS test to ensure their mental health. All participants filled out the consent form and completed the demographic questionnaires, which contained information on age, education, gender, and history of substance and alcohol use. Those with a history of substance and alcohol use were excluded. The Research Ethics Committee of Azad University approved this research (IR.IAU.SRB.REC.1397.044). Demographic data of the participants are presented in Table 1.

***Blood Samples and DNA Extraction***

Blood samples (5 mL of peripheral blood) were collected in EDTA tubes (Golden Vac., China). For DNA extraction, an extraction kit (Zhino Gene Pazhoohan, Iran) was used and performed according to the kit protocol. DNA specimens were stored at -20 °C.

***Using PCR-RFLP to detect rs13381800 and rs17039988 genotypes***

PCR amplification was done using the forward primer of 5'-GTATCTTGAAGCCCTGTGAGAA-3' and the reverse primer of 5'-GGTGCTTAAGAGCAAGTGAGA-3' for rs13381800 SNP in the promoter region of the TCF4 gene, and the forward primer of 5'-AGTGAAACCAGAAAGCTAGGG-3' and reverse primer of 5'-CAGAAATTATCGACTGGCACAAC-3' for rs17039988 SNP in the promoter region of the NRXN1 gene. 

Gene runner software (http://www.generunner.net/) and a website-based software called Oligo Analyzer (https://eu.idtdna.com/pages/tools/oligoanalyzer) were used to design the primers. The PCR reactions consisted of 2X buffer (CinnaGen, Iran), 10 mMdNTP of each base (CinnaGen, Iran) and 1.5 mMMgCl2 (CinnaGen, Iran), primers with a concentration of 10 pMOL, 2UTaq Polymerase (CinnaGen, Iran), and 50 Ng template DNA. The PCR thermal program for TCF4 and NRXN1 genes was conducted with initial denaturation at 94 94°C for 5 minutes, 40 cycles of 3 segments of the next denaturation at 94°C for 30 seconds, annealing at 55°C for 40 seconds for TCF4 and 54.5°C for NRXN1, followed by extension segment at 72°C for 40 seconds. The final extension was performed for 7 minutes at 72°C. The PCR products were run on 1.5% agarose gel and visualized by Green viewer on Doc UV illuminator. The DdeI enzyme was selected using NEBcutter database (http://nc2.neb.com/NEBcutter2/) and it could digest rs13381800 SNP in the presence of CC genotype that would cut the fragment into 3 bands with different sizes (190 bp, 49 bp, and 17 bp) (Table 2). The same process was also done for selecting the BsajI enzyme that could digest the rs17039988 SNP into two bands with different sizes (208bp and 134bp) in the presence of CC genotype.

A few samples were sequenced by the ABI capillary sequencing machine (FazaPazhooh Company, Iran) to verify genotypes of SNPs studied.

***Statistical Analysis***

SNP-STATS web application was used (https://www.snpstats.net/start.htm) to determine the frequency of alleles and genotypes. Also, data were analyzed using SPSS statistical software Ver.21.0. The association between SNP genotypes and schizophrenia was examined by χ2 test. Hardy-Weinberg equilibrium was examined by nonparametric tests (chi-squared test). The odds ratio and 95% CI were calculated by logistic regression. The association between genotypes frequencies and demographic and clinical traits was determined using chi-squared test, Fisher’s exact test, and likelihood ratio. Statistical significance was set at p <0.05.

## Results

**Figure 1:**
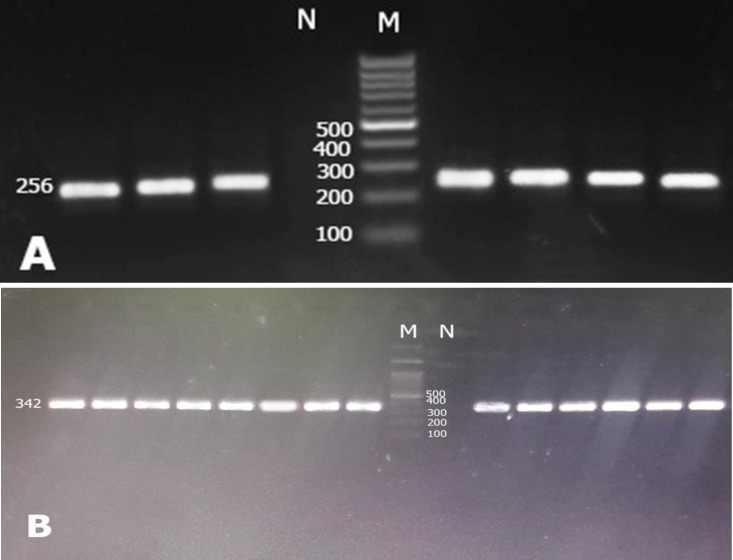
PCR Products of 2 Studied SNPs A) 256 bp Band for rs13381800 PCR product, B) 342 bp Band for rs17039988 PCR Product, M, 100bp Ladder, N, no DNA

**Figure 2:**
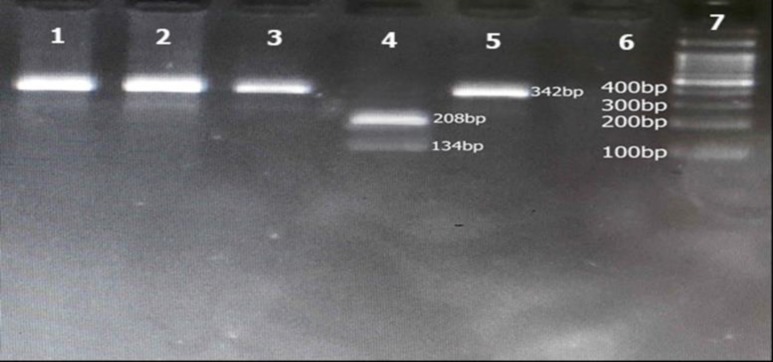
Digestion of PCR Products by BsajI No 1, 2, 3, and 5 Undigested and No 4 Digested Products. No 6, No DNA, and No 7, 50 bp DNA ladder

**Figure 3:**
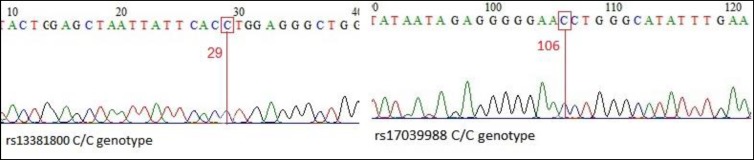
C/C Genotype of TCF4 rs13381800 and NRXN1 rs17039988 Single Nucleotide Polymorphisms (SNPs)

PCR product lengths were 256bp and 342 bp for TCF4 and NRXN1genes, respectively (Figure1). For enzyme digestion of SNP rs13381800, 0.1 μL (at a concentration of 10 units / μL) of the DdeI enzyme (Fermentase, Canada) at 37°C was used for 16 hours (Table 2). PCR product of NRXN1 gene for SNP rs17039988 was digested by BsajI (Fermentase, Canada): 0.2 μL (at a concentration of 10 units / μL) of the enzyme at 55°C for 16 hours. The digested products were run on 2% agarose gel containing 1 μL of Green Viewer 0.5 μg / mL (Pars Tous, Iran). The DdeI enzyme digestion produced 190bp, 49bp, and 17bp bands for CC genotypes and 256bp for TT genotype of rs13381800 SNP. For rs17039988 SNP, CC genotype produced two bands, including 208bp and 134 bp, while for AA genotype only 342 bp fragment was observed (Figure 2). Samples sequencing results are also shown in Figure 3.

The alleles and genotypes frequencies of rs13381800 in TCF4 and rs17039988 in NRXN1 are described in Table 3. For rs13381800, The OR value for CC genotype was 1.51 (0.61-3.71) with p-value = 0.366, indicating no statistically significant association between patients and controls. For rs17039988, only 16% of the population had CC genotype. The OR value for the CC genotype was 0.76 (0.27-2.13), which represented no remarkable difference between controls and patients with schizophrenia. No significant difference was found between the patients and the controls (P = 0.26). Since no heterozygotes were observed, the studied population was not in Hardy-Weinberg equilibrium.

A statistical analysis regarding demographic characteristics of the patients and controls and their association with the desired genotypes is presented in Table 4. The p-value of association between rs13381800 and gender was p = 0.912 for the patients and p = 0.635 for controls, respectively. The p-value of association between rs17039988 and gender was 0.484 for the patients and 0.418 for the controls. In addition, there was no significant difference in terms of age between rs13381800 genotypes in the patients (p = 0.675) and control groups (P = 1.000). No remarkable difference was observed between rs13381800 genotypes and education in the case group (p = 0.703) and in the healthy group (p = 0.345). Also, no association was found between rs17039988 genotypes and age in the patient (p = 0.374) and in the control groups (p = 1.000). No significant difference was observed between rs17039988 genotypes and education in the case group (p = 0.345) and control group (p = 0.588).

## Discussion

In the present study, it was hypothesized that there is a correlation between rs13381800 in the promoter region of the TCF4 gene and rs17039988 in the promoter region of the NRXN1 gene and schizophrenia. The results of the present study showed that these two polymorphisms were not associated with the incidence of schizophrenia. Since no heterozygote genotypes were observed for these two polymorphisms, the current sample of Iranian population was not in the Hardy-Weinberg equilibrium. We could not obtain a hereditary model for these alleles, and this imbalance could be due to the small population of our study and to the heterozygote disadvantage, as the nonmutant allele is more compatible to survive than mutant allele (37). Males are more likely to develop this mental disorder, but in the present study no significant difference was observed between gender and both SNPs in the case group (2). Scholastic achievement has an inverse relationship with the risk of developing schizophrenia, but in this study there was no meaningful difference between education and SNPs in the case and groups (3).

In the present study, for rs13381800, the frequency of the CC genotype, which had a mutant allele, was 9%. For rs17039988, in the promoter region of the NRXN1 gene, the CC genotype had a mutant allele of 9%. The results of similar surveys for Africa (AFR), America (AMR), East Asia (EAS), Europe (EUR), and South Asia (SAS) were given in the NCBI database (https://www.ncbi.nlm.nih.gov/ projects / SNP). As shown in Table 5, for rs13381800 in the promoter region of the TCF4 gene, there was a C allele in all populations, and it was more abundant in the East Asian population than in others. Regarding the frequencies reported on

NCBI database for rs17039988 in the promoter region of the NRXN1 gene, the allele frequency observed in this study for alleles A and C was higher than the frequency reported for other populations. The reason for this difference may be genetic differences. 

**Table 1:** Demographic Characteristics of Patients with Schizophrenia and Healthy Groups

Variables	Case	Control
Continuous Variable		
Age	40.80 ± 11.298	32.92 ± 7.391
Discontinuous Variables		
Gender		
Female	35 (35%)	37 (37%)
Male	65 (65%)	63 (63%)
Educational Status		
Illiterate	10 (10%)	0 (0%)
Elementary	42 (42%)	4 (4%)
High School Bachelor Masters PhD	34 (34%) 12 (12%) 1 (1%) 1 (1%)	13 (13%) 35 (35%) 39 (39%) 9 (9%)

**Table 2:** Sequences of the Primers Used for PCR in the Current Study

**Polymorphism**	**Sequences of primers**	**Restriction enzymes**	**Length (bp)**
	Forward Primer:		
rs13381800	5'-GTATCTTGAAGCCCTGTGAGAA-3'		TT (256)
	Reverse Primer:	DdeI	TC (256+190+49+17)
	5'-GGTGCTTAAGAGCAAGTGAGA-3'		CC (190+49+17)
			
	Forward Primer:		
rs17039988	5'-AGTGAAACCAGAAAGCTAGGG-3'		AA (342)
	Reverse Primer:	BsajI	AC (342+208+134)
	5'- CAGAAATTATCGACTGGCACAAC-3'		CC (208+134)

PCR; Polymerase chain reaction, bp; Base pair

**Table 3:** Frequency of the Genotype of rs13381800 and rs17039988 in the Healthy Individuals Group and Patients with Schizophrenia Group

	**Genotype n (%)** **TT CC ** ***χ*** ^2^ ** P-value**	**Allele n (%)** **T C χ2 P-value**	**OR**	**95% CI**
rs13381800				
Case	91 9 (91%) (9%)	182 18 (91%) (9%)		
Control	87 13 (87%) (13%)	174 26 (87%) (13%)		
	0.8172 0.366012	1.6343 0.201106	1.51	(0.61-3.71)
	AA CC *χ*^2^ P-value	A C *χ*^2^ P-value		
rs17039988				
Case	91 9 (91%) (9%)	182 18 (91%) (9%)		
Control	93 7 (93%) (7%)	186 14 (93%) (7%)		
	0.2717 0.602168	0.5435 0.460995	0.76	(0.27-2.13)

**Table 4:** The Association between rs13381800 and rs17039988 Genotypes with Demographic Characteristics of Patients and Healthy Individuals

	**rs13381800**			**rs17039988**		
Parameters	CC	TT	P-value	AA	CC	P-value
Gender						
Case						
Female	3%	32%		33%	2%	
Male	6%	59%		58%	7%	
			0.912			0.488
Control						
Female	4%	33%		33%	4%	
Male	9%	55%		61%	3%	
			0.635			0.418
Age						
Case						
20 - 30	1%	19%		14%	2%	
31 - 40	5%	35%		34%	4%	
41 - 50	1%	18%		19%	0%	
51 - 60	1%	16%		19%	3%	
61 - 70	1%	2%		4%	0%	
71 - 80	0%	1%		1%	0%	
			0.675			0.374
Control						
20 - 30	0%	1%		1%	0%	
31 - 40	13%	86%		92%	7%	
41 - 50	0%	0%		0%	0%	
51 - 60	0%	0%		0%	0%	
61 - 70	0%	0%		0%	0%	
71 - 80	0%	0%		0%	0%	
			1.000			1.000
Educational Status						
Case						
Illiterate	0%	10%		9%	1%	
Elementary	4%	38%		39%	3%	
High School	3%	31%		33%	1%	
Bachelor	2%	10%		8%	4%	
Masters	0%	1%		1%	0%	
PhD	0%	1%		1%	0%	
			0.703			0.345
Control						
Illiterate	0%	0%		0%	0%	
Elementary	2%	2%		4%	0%	
High School	1%	12%		13%	0%	
Bachelor	4%	31%		32%	3%	
Masters	4%	35%		36%	3%	
PhD	2%	7%		8%	1%	
			0.345			0.588

**Table 5:** The rs17039988 and rs13381800 Allele Frequencies Observed in Different Populations

Submitted SNP(ss) : ss1297143493 Handle : 1000 GENOMES Project RefSNP : rs17039988			
Population	Chromosome Sample Count	Alleles	
		A	C
EAS	1008	1.000	0.000
EUR	1006	1.000	0.000
AFR	1322	0.906	0.093
AMR	694	0.994	0.005
SAS	978	1.000	0.000
Submitted SNP(ss) : ss1361245306 Handle : 1000 GENOMES Project RefSNP : rs13381800			
Population	Chromosome Sample Count	Alleles	
		T	C
EAS	1008	0.692	0.307
EUR	1006	0.697	0.302
AFR	1322	0.904	0.095
AMR	694	0.783	0.216
SAS	978	0.879	0.120

EAS; East Asian, EUR; European, AFR; African, AMR; East Asian, SAS; South Asian

## Limitation

Convincing the patients especially those with paranoia for completing written consent was the limitation of this study.

## Conclusion

In conclusion, depending on the collected data, it was revealed that rs13381800 SNP in TCF4 gene and rs17039988 SNP in NRXN1 gene were not risk factors for schizophrenia in this sample of Iranian patients with schizophrenia. Also, no significant differences were observed between the genotypes for rs13381800 and rs17039988 and age, gender, and education in case and healthy groups. However, more studies should be conducted with large sample size and on various populations to clarify the effect of these SNPs on the risk of schizophrenia.
